# Microscopic Polyangiitis With Temporal Artery Involvement Mimicking Giant Cell Arteritis

**DOI:** 10.7759/cureus.110271

**Published:** 2026-06-04

**Authors:** Janhvi Shah, Keith A Bernstein

**Affiliations:** 1 Internal Medicine, St. Luke’s Hospital, Chesterfield, USA; 2 Rheumatology, St. Luke's Hospital, Chesterfield, USA

**Keywords:** anca-associated vasculitis, anti-myeloperoxidase antibodies, avacopan, giant cell arteritis, microscopic polyangiitis, rituximab, temporal arteritis

## Abstract

A 63-year-old woman presented with a four-month history of bilateral hearing loss and three months of progressive, painful peripheral neuropathy. Her clinical course was complicated by deep vein thrombosis with pulmonary emboli and an incomplete response to initial empiric glucocorticoid therapy. Following glucocorticoid taper, she developed temporal headaches, jaw claudication, and scalp tenderness, raising concern for giant cell arteritis (GCA). Laboratory evaluation revealed markedly elevated inflammatory markers (erythrocyte sedimentation rate and C-reactive protein), microcytic anemia, mild hematuria, and strongly positive myeloperoxidase-antineutrophil cytoplasmic antibody (MPO-ANCA) by antigen-specific immunoassay with negative proteinase-3 (PR3)-ANCA. Temporal artery biopsy demonstrated transmural inflammation affecting all vessel layers without giant cells or granulomata. The combination of multisystem small-vessel involvement, MPO-ANCA seropositivity with negative PR3-ANCA, and characteristic histopathological findings established the diagnosis of microscopic polyangiitis (MPA) with temporal arteritis rather than granulomatosis with polyangiitis (GPA) or GCA. Treatment with high-dose glucocorticoids (prednisone 80 mg daily), avacopan (30 mg twice daily), and rituximab (375 mg/m² weekly for four weeks) resulted in rapid improvement of constitutional and cranial symptoms. This case highlights the importance of integrating MPO-ANCA serology and histopathological findings when temporal arteritis is the presenting feature of systemic vasculitis. It illustrates the utility of avacopan-based, glucocorticoid-sparing combination immunosuppression in this context.

## Introduction

Microscopic polyangiitis (MPA) is a systemic pauci-immune necrotizing vasculitis predominantly affecting small vessels, including arterioles, capillaries, and venules, and is most commonly associated with myeloperoxidase-antineutrophil cytoplasmic antibody (MPO-ANCA) positivity [1]. MPA is one of three major subtypes of ANCA-associated vasculitis (AAV), alongside granulomatosis with polyangiitis (GPA) and eosinophilic GPA (EGPA). While renal involvement (rapidly progressive glomerulonephritis) and pulmonary capillaritis are the most frequent organ manifestations, peripheral neuropathy, upper airway disease, and ocular involvement are well described [1,2].

Temporal artery involvement in AAV is uncommon but clinically significant, as it may closely mimic giant cell arteritis (GCA), the most common primary large-vessel vasculitis in adults over 50 years of age. Both conditions can present with temporal headache, jaw claudication, and elevated inflammatory markers, making clinical distinction challenging [3,4]. Histopathologically, GCA classically demonstrates granulomatous inflammation with multinucleated giant cells and disruption of the internal elastic lamina, whereas AAV-related temporal arteritis more often shows non-granulomatous transmural inflammation with lymphoplasmacytic and neutrophilic infiltrates in the setting of systemic small-vessel disease [3,4]. However, the absence of giant cells does not exclude GCA, and accurate diagnosis requires integration of histopathology, serological testing (particularly ANCA specificity), and the broader clinical context.

We report a case of MPO-ANCA-positive MPA presenting with temporal artery involvement initially concerning for GCA, illustrating how AAV can mimic GCA. This case also highlights the role of rituximab-based induction therapy and the evolving place of avacopan as a glucocorticoid-sparing agent in multisystem disease.

## Case presentation

A previously healthy 63-year-old woman presented with four months of bilateral hearing loss and three months of progressive, painful distal sensory symptoms. Paresthesias began in the proximal lower extremities and progressed distally to the feet and hands, with associated weakness impairing ambulation and requiring a walker. Concurrent sinonasal congestion and intermittent epistaxis were noted.

She subsequently developed bilateral lower extremity deep vein thrombosis and pulmonary emboli, treated with apixaban. Hypercoagulability workup revealed a prothrombin gene mutation. Empiric prednisone (40 mg daily) led to transient improvement followed by relapse during taper. Following taper, she developed new temporal headaches, scalp tenderness, and jaw claudication with weight loss (~4.5 kg). Examination showed 4/5 bilateral lower extremity weakness, stocking-glove sensory loss, and right sixth cranial nerve palsy. No temporal artery tenderness was present.

Inflammatory markers were markedly elevated (Table 1). Serological evaluation confirmed strongly positive MPO-ANCA by antigen-specific immunoassay (Table 1).

**Table 1:** Laboratory values. MPO-ANCA: myeloperoxidase-antineutrophil cytoplasmic antibody; PR3: proteinase-3

Test	Observed value	Reference range
Erythrocyte sedimentation rate (mm/hr)	100	0-20
C-reactive protein (mg/L)	21.7	<5.0
Hemoglobin (g/dL)	9.4	12.0-16.0
Mean corpuscular volume (fL)	81.8	80.0-100.0
Urinalysis: RBCs per high-power field	6-10	0-2
Serum creatinine (mg/dL)	0.72	0.50-1.10
MPO-ANCA (antigen-specific immunoassay)	Strongly positive	Negative
PR3-ANCA (antigen-specific immunoassay)	Negative	Negative
Rheumatoid factor (IU/mL)	116.6	<14.0

**Figure 1:**
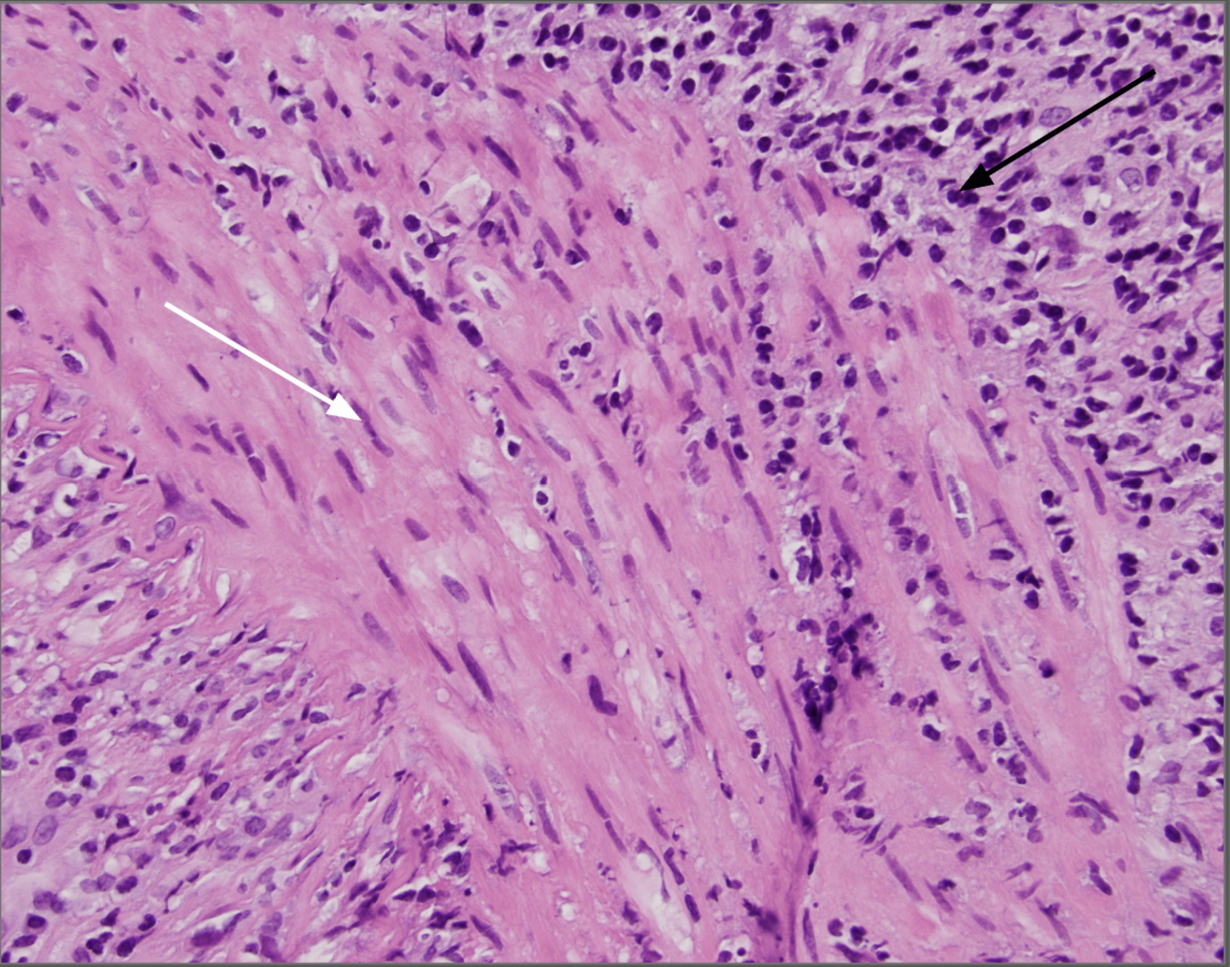
400x magnification (H&E stain) of the temporal artery wall showing transmural inflammation. Black arrow: dense lymphoplasmacytic and neutrophilic infiltrate throughout the arterial wall. White arrow: plasma cells within the inflammatory infiltrate. Multinucleated giant cells are absent.

**Figure 2:**
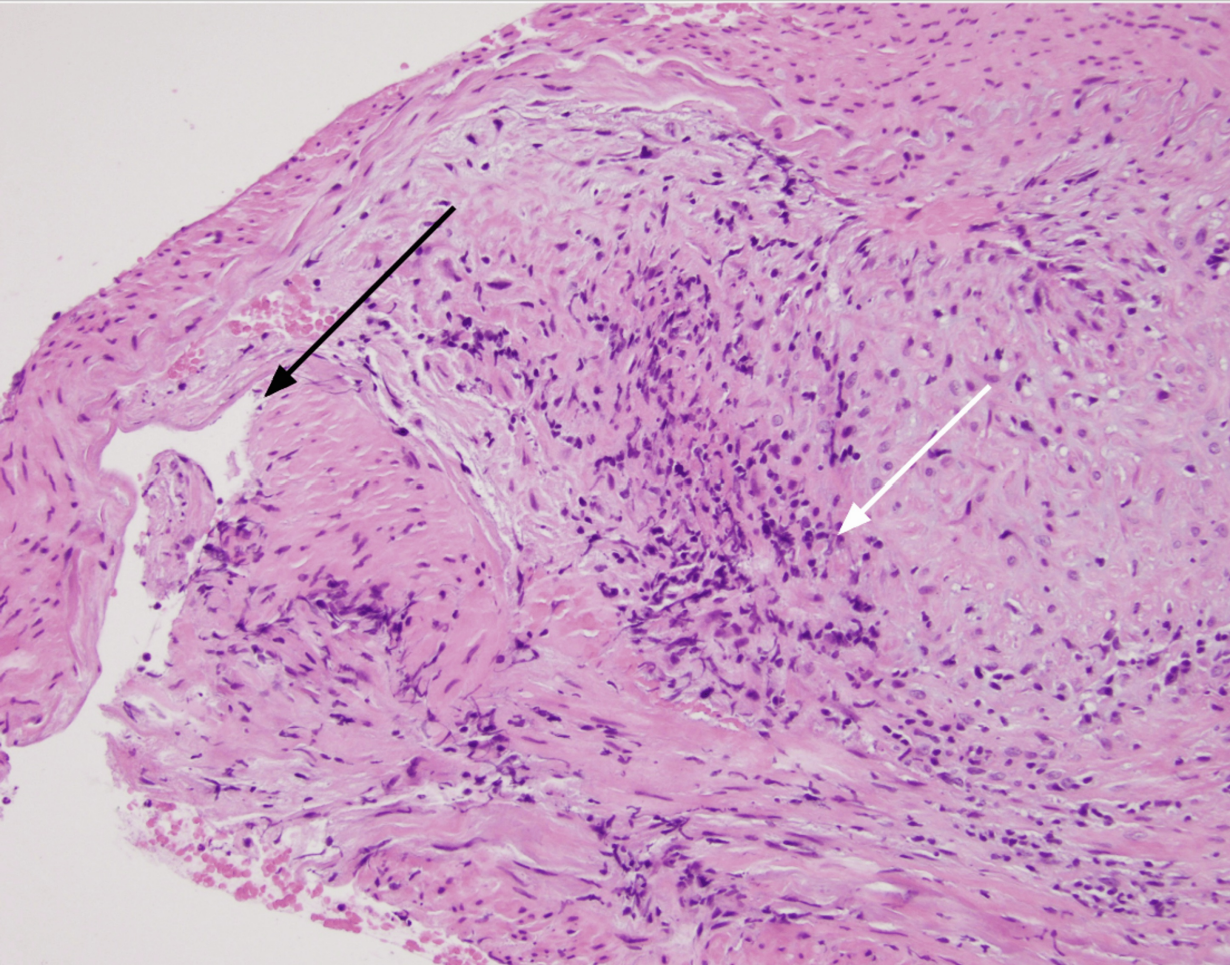
200x magnification (H&E stain) showing near-complete luminal occlusion. Black arrow: markedly narrowed arterial lumen (approximately 80% stenosis). White arrow: thickened intima with inflammatory hyperplasia. No granulomata or giant cells are identified. Necrobiosis is visible.

**Figure 3:**
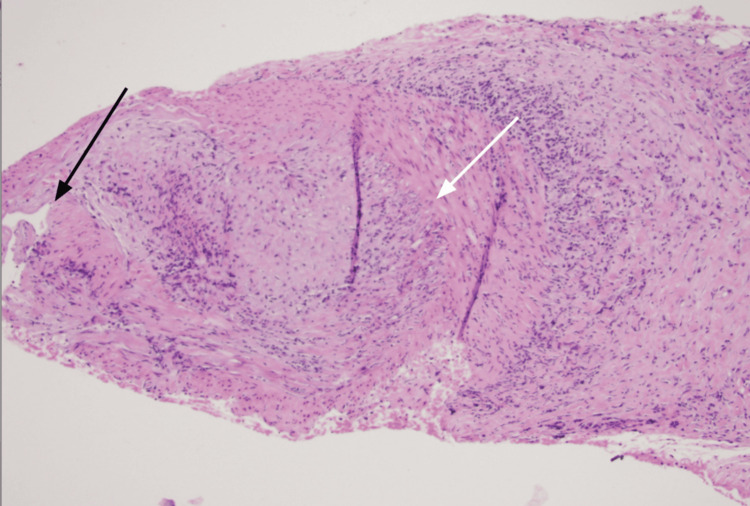
100x magnification (H&E stain) showing the affected vessel segment. Black arrow: near-complete luminal occlusion by inflammatory infiltrate. White arrow: intimal hyperplasia contributing to luminal narrowing.

**Figure 4:**
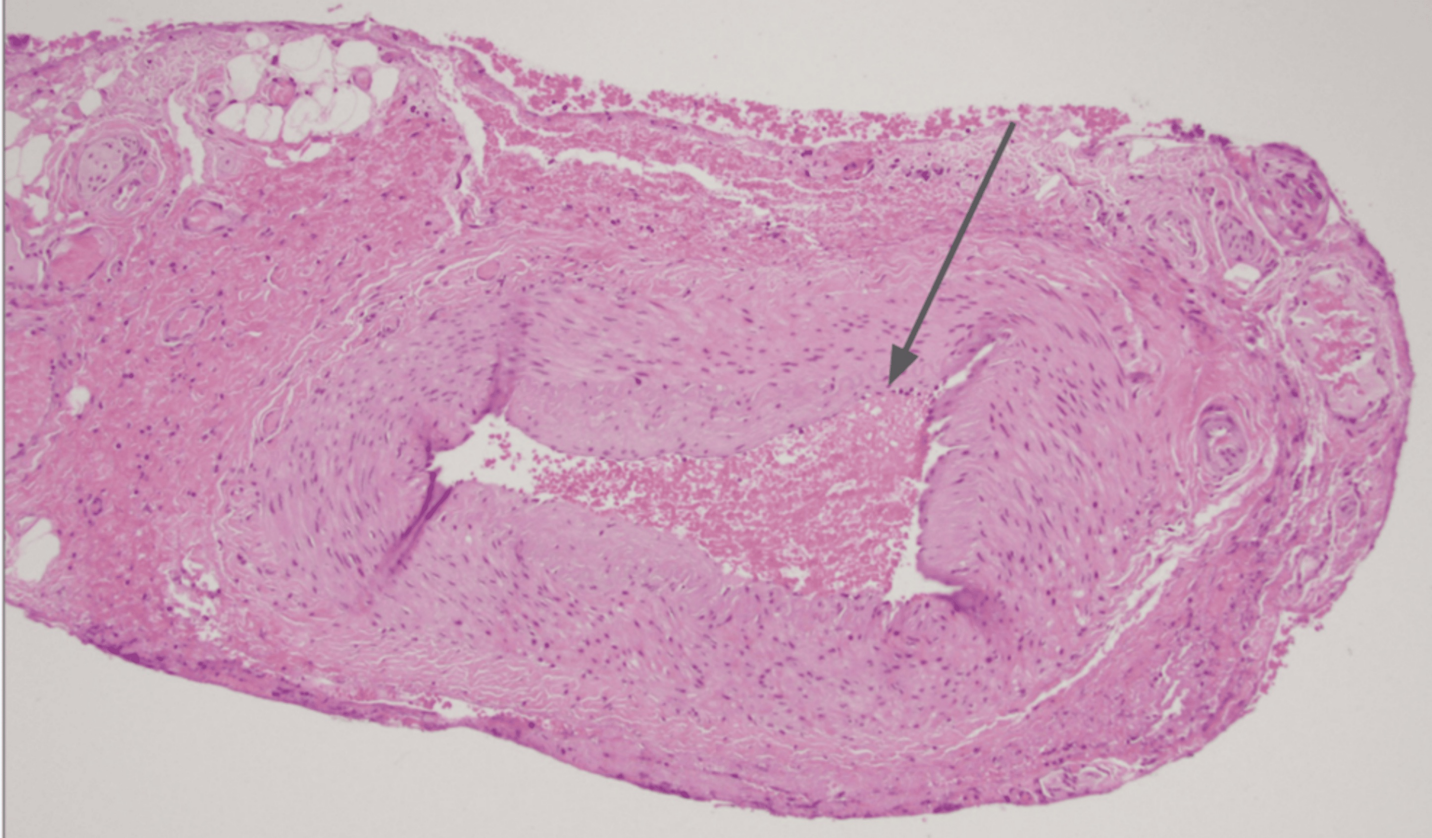
100x magnification (H&E stain) from the same biopsy specimen showing a normal vessel segment adjacent to the inflamed segment. Black arrow: patent arterial lumen without inflammation. This skip-lesion pattern, focal involvement with intervening normal segments, is characteristic of vasculitis.

Transmural inflammation with lymphoplasmacytic and neutrophilic infiltrates, intimal hyperplasia, and approximately 80% luminal narrowing are demonstrated at 400x (Figure 1), 200x (Figure 2), and 100x magnification (Figure 3). To be noted, it is the same slide figure captured at different magnifications (Figure 1 shows 400x, Figure 2 shows 200x, and Figure 3 shows 100x). A skip lesion, a normal uninflamed vessel segment immediately adjacent to the affected segment, is shown at 100x in Figure 4. Overall findings favored MPO-ANCA-associated MPA with temporal artery involvement over GCA or GPA.

The Five-Factor Score (FFS), a validated prognostic tool for systemic necrotizing vasculitides, was calculated for this patient using both the original 1996 and revised 2009 criteria [5,6]. The 1996 FFS was 0 (no proteinuria > 1 g/day, renal insufficiency, gastrointestinal involvement, cardiomyopathy, or definite central nervous system involvement), and the 2009 FFS was 0 (age 65, no cardiac or renal insufficiency, no gastrointestinal involvement, and with a protective point for ENT manifestations). Despite a low FFS, aggressive induction therapy with high-dose prednisone (80 mg daily), rituximab (375 mg/m² weekly ×4), and avacopan (30 mg twice daily) was initiated given the presence of severe, organ-threatening manifestations, including biopsy-proven temporal arteritis with ~80% luminal narrowing, progressive mononeuritis multiplex with motor impairment, and cranial neuropathy, which are not fully captured by the FFS but warrant intensive immunosuppression. Following remission, the patient was transitioned to rituximab maintenance therapy with continued avacopan and glucocorticoid taper, consistent with KDIGO (Kidney Disease: Improving Global Outcomes) 2024 and ACR (American College of Rheumatology)/Vasculitis Foundation recommendations. Apixaban was temporarily held and resumed after one week.

The diagnosis of MPA was confirmed using the 2022 ACR/European Alliance of Associations for Rheumatology (ACR/EULAR) classification criteria for MPA. These criteria apply a weighted scoring system to patients with an established diagnosis of small- or medium-vessel vasculitis after exclusion of mimics; a cumulative score of ≥5 classifies a patient as having MPA (sensitivity 91%, specificity 94%). Application of these criteria to the present case yielded a total score of +3, as detailed in Table 2.

**Table 2:** Application of the 2022 ACR/EULAR classification criteria for microscopic polyangiitis (MPA) [7]. Classification threshold: ≥5 points = MPA p-ANCA: perinuclear antineutrophil cytoplasmic antibody; MPO-ANCA: myeloperoxidase-antineutrophil cytoplasmic antibody; c-ANCA: cytoplasmic antineutrophil cytoplasmic antibody; PR3: proteinase-3; ACR: American College of Rheumatology; EULAR: European Alliance of Associations for Rheumatology

Criterion	Weight	Present in this patient	Score
p-ANCA or anti-MPO-ANCA positivity	+6	Yes (strongly positive MPO-ANCA)	+6
Pauci-immune glomerulonephritis on biopsy	+3	No (renal biopsy not performed)	0
Pulmonary fibrosis or interstitial lung disease	+3	No	0
Sinonasal symptoms or signs	−3	Yes (sinonasal congestion, epistaxis)	−3
c-ANCA or anti-PR3-ANCA positivity	−1	No (PR3-ANCA negative)	0
Eosinophil count ≥ 1 × 10⁹/L	−4	No	0
Total score			+3

The patient's total score of +3 falls below the classification threshold of ≥5, primarily because sinonasal symptoms, which carry a negative weight (−3) in the MPA criteria, as they are more characteristic of GPA, offset the strongly positive MPO-ANCA (+6). However, it is important to note that the 2022 ACR/EULAR criteria are classification criteria designed for clinical research cohorts, not diagnostic criteria. The diagnosis of MPA in this case was established clinically based on the constellation of strongly positive MPO-ANCA, biopsy-proven small-vessel vasculitis of the temporal artery with pauci-immune transmural inflammation (absence of granulomata and giant cells), microscopic hematuria, peripheral neuropathy, and cranial neuropathy, features consistent with MPA rather than GPA or GCA. The 2022 ACR/EULAR criteria themselves acknowledge that ANCA specificity is assigned the highest weight and that clinical, histological, and serological features should be integrated for diagnosis.

Rapid improvement in constitutional and cranial symptoms occurred within one week, while neuropathy improved more gradually. At three months, hematuria resolved, and neurological symptoms partially improved. She transitioned to rituximab maintenance therapy with continued avacopan and steroid taper.

## Discussion

This case highlights the diagnostic challenge of AAV presenting with temporal artery involvement, a rare manifestation that can closely mimic GCA. The key challenge was distinguishing MPA from GCA and GPA in the setting of overlapping cranial, neurologic, and ENT features.

Temporal artery involvement in AAV is uncommon but well described and may clinically resemble GCA [3,8,9]. Although the absence of giant cells and granulomas may support AAV, these findings are not independently diagnostic, as giant cells may be absent in a substantial proportion of biopsy-proven GCA cases. In this case, isolated GCA was considered less likely given multisystem involvement, MPO-ANCA positivity, and non-granulomatous neutrophilic arteritis. However, concurrent large-vessel GCA cannot be fully excluded without vascular imaging.

GPA was considered due to sinonasal symptoms and hearing loss. However, MPO-ANCA positivity with PR3 negativity and absence of granulomatous inflammation favored MPA, although MPO-positive GPA remains a recognized entity [10]. Lack of sinus imaging remains a limitation.

Antigen-specific ANCA testing was critical, as immunofluorescence patterns (c-ANCA and p-ANCA) were discordant with antigen-specific results. Current guidelines recommend antigen-specific MPO/PR3 testing as the diagnostic standard in suspected AAV [7,11].

Treatment with rituximab, glucocorticoids, and avacopan is consistent with guideline-supported induction therapy [12]. Rituximab efficacy for remission induction in AAV was established in the RAVE and RITUXVAS trials [13,14]. Avacopan, an oral C5a receptor antagonist, was included in this patient's regimen based on the glucocorticoid-sparing potential demonstrated in the ADVOCATE trial, in which avacopan was non-inferior to prednisone taper for remission at week 26 and superior for sustained remission at week 52 [15]. However, the regulatory status of avacopan has since become uncertain: the FDA/Center for Drug Evaluation and Research (CDER) has raised concerns regarding the integrity of the ADVOCATE efficacy data, specifically that non-blinded personnel reportedly re-adjudicated certain endpoints after database lock, converting an initially non-significant sustained remission analysis into a significant result-findings that were not properly disclosed to the agency. Additionally, post-marketing surveillance has identified serious hepatotoxicity as an important safety signal, including cases of severe drug-induced liver injury and vanishing bile duct syndrome, some of which were fatal [16,17]. These developments have prompted the FDA to request withdrawal of approval for Tavneos® (avacopan). In the present case, the patient tolerated avacopan without hepatic complications, and clinical improvement occurred within the context of a multimodal regimen including high-dose glucocorticoids and rituximab, making it difficult to attribute the response to any single agent.

Neuropathy likely reflects ischemic injury to the vasa nervorum and often improves incompletely [18,19]. Hearing loss is a recognized but less common manifestation of AAV [20].

## Conclusions

Temporal artery involvement in AAV may closely mimic GCA and should be considered in patients with cranial symptoms and systemic features of small-vessel vasculitis. Diagnosis requires integration of serologic, histopathologic, and clinical findings rather than reliance on any single feature. In this case, the overall clinicopathologic picture favored MPA over GCA or GPA. Early recognition and prompt initiation of rituximab-based therapy remain central to improving outcomes in AAV while minimizing glucocorticoid exposure. The role of avacopan in this paradigm is now uncertain, given the FDA's concerns regarding ADVOCATE trial data integrity and emerging post-marketing hepatotoxicity signals. Looking ahead, the AAV therapeutic landscape is evolving rapidly: next-generation anti-CD20 monoclonal antibodies such as obinutuzumab have shown promising efficacy in refractory disease, novel complement pathway inhibitors are under investigation, and anti-CD19 chimeric antigen receptor (CAR) T-cell therapy has demonstrated drug-free remission in early case reports of refractory AAV. These emerging approaches may offer more durable remission with reduced treatment-related toxicity, though further prospective studies are needed to define their optimal role.
